# A Convolutional Neural Network Face Recognition Method Based on BiLSTM and Attention Mechanism

**DOI:** 10.1155/2023/2501022

**Published:** 2023-01-19

**Authors:** Xiaobo Qi, Chenxu Wu, Ying Shi, Hui Qi, Kaige Duan, Xiaobin Wang

**Affiliations:** ^1^School of Computer Science and Technology, Taiyuan Normal University, Jinzhong 030619, China; ^2^School of Computer and Information Technology, Shanxi University, Taiyuan 030006, China

## Abstract

Face recognition technology is a powerful means to capture biological facial features and match facial data in existing databases. With the advantages of noncontact and long-distance implementation, it is being used in more and more scenarios. Affected by factors such as light, posture, and background environment, the face images captured by the device are still insufficient in the recognition rate of existing face recognition models. We propose an AB-FR model, a convolutional neural network face recognition method based on BiLSTM and attention mechanism. By adding an attention mechanism to the CNN model structure, the information from different channels is integrated to enhance the robustness of the network, thereby enhancing the extraction of facial features. Then, the BiLSTM method is used to extract the timing characteristics of different angles or different time photos of the same person so that convolutional blocks can obtain more face detail information. Finally, we used the cross-entropy loss function to optimize the model and realize the correct face recognition. The experimental results show that the improved network model indicates better identification performance and stronger robustness on some public datasets (such as CASIA-FaceV5, LFW, MTFL, CNBC, and ORL). Besides, the accuracy rate is 99.35%, 96.46%, 97.04%, 97.19%, and 96.79%, respectively.

## 1. Introduction

Face recognition technology is to extract facial features from the input of face images and then realize the function of face recognition. Face recognition technology has been one of the hottest research topics in recent years. It combines many specialized technologies and has been extensively researched and developed. The principle of face recognition technology mainly contains four parts: the acquisition and preprocessing of face images, face detection, face feature extraction, and face recognition. Face detection is mainly used to mark the location and size of faces in images. The extraction of facial features is to model the extracted facial features. In modern society, face recognition technology has been widely used in government, education, and other fields, such as access control, ATM, and attendance. Face recognition technology [1] has attracted much attention.

The rapid development of deep learning has made face recognition technology more mature. Since the temporal characteristics of real pictures of human faces will be affected by factors such as illumination, posture, and background environment, the face recognition technology will be inaccurate. Face recognition technology is not well applied in the case of recognizing multiple face pictures of the same person in a short time. For example, the CASIA-FaceV5 dataset includes 2,500 images of 500 people, with an average of 5 different images per person with the same background. The classic deep learning-based face recognition method does not consider the timing characteristics between different photos of the same person. Therefore, the recognition efficiency is low. This paper considers the temporal and geometric features of different face images. A convolution neural network face recognition method based on BiLSTM and attention mechanism called AB-FR is proposed.

The model proposed in this paper has the following contributions:

1. The proposed model is based on CNNs, and an attention mechanism is added to extract important face block features and better process face feature information
2. The BiLSTM (bidirectional long short-term memory) is added to the proposed model to capture the sequence features of the image and extract the hidden temporal features, so as to further improve the accuracy of feature selection
3. The cross-entropy is used as a loss function to classify the data, thus improving training efficiency and classification accuracy

The rest of this paper is organized as follows. Section 2 describes the related work. Section 3 briefly introduces some background knowledge about CNN, BiLSTM, and SENet. Section 4 presents our AB-FR model in detail and describes our experimental results and discussion. Section 5 concludes the paper.

## 2. Related Work

Traditional face recognition techniques include facial recognition based on geometric features [2], facial recognition based on feature faces [3], and methods based on hidden Markov models [4]. In face recognition based on geometric features, geometric features can be eyes, nose, mouth, and other shapes and geometric relationships between them (such as their distance from each other). Yuangen et al. [2] used the AdaBoost algorithm combining skin color detection and geometric features, which uses skin color to roughly filter face candidate regions. However, face detection based on skin color is sensitive to light and noise environments, which makes the face detection effect unsatisfactory.

The face recognition method based on feature face reflects the information hidden in the face sample set and the structural relationship of the face. It is easily affected by factors such as facial expression, posture, and lighting and has great limitations. Wang et al. [3] proposed an image processing method based on the fusion of facial key detection points and grayscale transformation. It reduces the complexity of face recognition by adjusting the saliency, contrast, and data complexity of face image features. However, when using low-resolution images, due to their low feature information and high sensitivity to image occlusion, this method retains insufficient features and has a low recognition rate.

In the face recognition method based on the hidden Markov model (HMM), the hidden Markov model is a probabilistic model about time series, which can extract the face image observation sequence. Each area of the face has multiple states, through which the HMM of the face image can be established. Wang and Liu [4] used the facial features extracted from the hidden Markov model and the characteristics of the Viterbi algorithm to segment the facial feature sequence. But it does not consider the correlation between sequence annotation and the length and context of the observed sequence, so the correct rate of face recognition is not high enough.

In recent years, deep learning [5] has been widely used, especially in the face recognition field. It is different from the traditional methods. The face recognition method based on deep learning is more inclusive in face feature selection and has strong adaptability to the influence of lighting and changes in expressions. Common face recognition methods based on deep learning include MTCNN [6], DeepID [7], FaceNet [8], and DeepFace [9].

MTCNN [6] adopts the thought of the cascade, including P-Net, R-Net, and O-Net three-layer networks. It uses the image pyramid algorithm, which needs to scale the image several times, resulting in a large number of forwarding operations, which seriously slows down the detection speed. FaceNet [8] is a deep convolutional neural network. At the heart of this method are millions of training data and Triplet-Loss. Therefore, the model requires a lot of calculation and runs slowly. DeepFace [9] improves the face alignment method using explicit 3D face modeling and piecewise affine transformation applied to frontal faces. But the model has to be rebuilt with each call, resulting in a very high memory requirement.

It can be seen from the above that a convolutional neural network is a commonly used deep learning method in face recognition. It has good self-organization and adaptive abilities and can implicitly describe many laws of face recognition through the learning process. It is more adaptable, but it also has some drawbacks that make it less effective.

Tao et al. [10] proposed the combination of CNN and metric learning methods for face recognition, which uses a multi-Inception structure to extract facial features. But it requires too much data sample. Hu et al. [11] fused the extracted multi-layer features in the subspace, further improving the face recognition effect. However, the number of parameters is too large and the operation is slow. Wu and Zhang [6] changed the SoftMax layer in MobileNet to L-SoftMax to avoid overfitting and achieve better classification results. But it deepens the network and increases the running time, which is not efficient. Ren and Xue [12] proposed an R-CNN face recognition method, which combines ResNet with CNN. However, the scale of the network is too large and requires the support of a large number of datasets.

## 3. Method of AB-FR

In this section, our network is based on CNN structure and we add BiLSTM to extract the bidirectional time series features of the images. Then, an attention mechanism is added to extract the important features of the images. Finally, the AB-FR model is formed.

### 3.1. Convolutional Neural Networks

**Figure 1:**
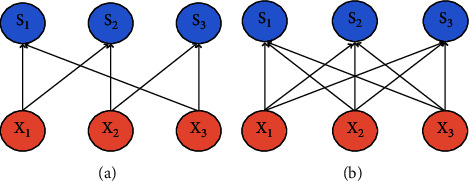
The difference between local connection and full connection. (a) Local connection. (b) Full connection.

**Figure 2:**
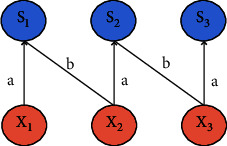
Weight sharing.

**Figure 3:**
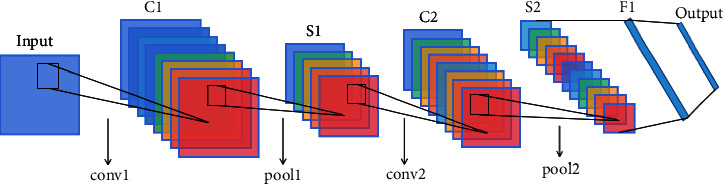
Convolutional neural network model.

The underlying network structure used in this paper is CNN (convolutional neural network) [13–16]. It is a feedforward neural network that avoids complex image preprocessing. The difference between convolutional neural networks and general neural networks is that the local connection and weight sharing reduce the number of weights to be trained, thereby reducing the learning complexity of the network model.  Figure 1(a) shows the local connection, and Figure 1(b) shows the full connection.  Figure 2 represents weight sharing. The structure of the convolutional neural network includes a convolutional layer, a pooled layer, and a fully connected layer. Its classic model is shown in Figure 3.

### 3.2. BiLSTM

The CASIA-FaceV5 dataset used in this paper includes images of 500 people, and each person contains five different images of the same background. The LFW dataset has a total of 5,749 people, including 13,233 face images, and some of them have more than two images. The MTFL dataset contains nearly 13,000 face images from the Internet. The CNBC dataset collects mugshots of 200 people from various states. The ORL dataset includes the faces of 40 people, each with 10 images. There must be a temporal relationship between images of different periods or different angles of each person, so we add BiLSTM to the network model. In this way, the time series information between different images can be fully considered, so that the time series feature vector of the face image can be obtained and sent to the attention mechanism.

**Figure 4:**
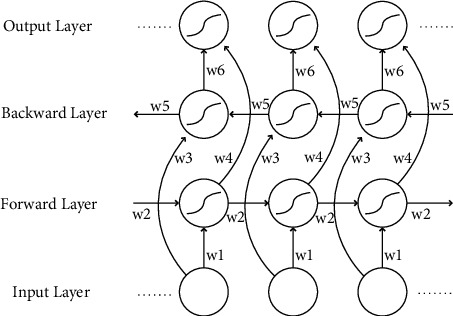
BiLSTM network structure diagram.

Long short-term memory (LSTM) network [17] is a time series convolutional neural network derived from a recurrent neural network. By introducing a gate function, it is possible to mine the time series variation laws of relatively long intervals and delays in time series. The bidirectional long short-term memory (BiLSTM) network consists of a forward LSTM network and a backward LSTM network, and its network structure is shown in Figure 4.

Among them, *w*_1_ − *w*_6_ represents six shared weights, which are calculated forward in the forward layer, and the output *h*_*t*_ of the forward hidden layer from time 1 to time *t* is obtained and saved. In the backward layer, a reverse calculation is performed to obtain the output *h*_*t*_′ of the backward hidden layer from time *t* to time 1, and it is saved. Finally, combined with the output result of the corresponding moment of the forward layer and the backward layer, the final output *O*_*t*_ is obtained, and its expression is as follows:

(1) $$\begin{array}{c} h_{t}=f\left(w_{1}x_{t}+w_{2}h_{t-1}\right),\\ h_{t}^{\prime }=f\left(w_{3}x_{t}+w_{5}h_{t+1}^{\prime }\right),\\ O_{t}=g\left(w_{4}h_{t}+w_{6}h_{t}^{\prime }\right). \end{array}$$

By adding BiLSTM, this paper extracts the bidirectional sequence features based on images and automatically generates the context relationship of the sequence, which effectively increases the amount of information available to the network model, improves the context information available to the algorithm, and improves the accuracy of face recognition.

### 3.3. Attention Mechanism Module

Attention mechanism [18] is a data processing method in machine learning. Generally speaking, when people observe external things, they tend to observe some important local information of things first. Then, information from different regions is combined to form an overall impression of what is being observed.

This paper adopts the typical channel attention mechanism SENet to capture more important feature information, and its calculation is mainly divided into two steps.

*Step 1*. Squeeze operation: perform a global average pooling operation on the input features and compress the *H* × *W* × *C* features into a 1 × 1 × *C* size.
  (2) $$\begin{array}{c} Z_{c}={\text{F}}_{\text{sq}}\left(u_{c}\right)\\ =\frac{1}{H\times W}\overset{H}{\underset{i=1}{\sum }}\overset{W}{\underset{j=1}{\sum }}u_{c}\left(i,j\right). \end{array}$$
*Step 2*. Excitation operation: perform two full connection operations on the result of the squeeze operation and then use sigmoid activation to obtain the weight matrix.

(3) $$\begin{array}{c} S=F_{\text{ex}}\left(z,W\right)\\ =\sigma \left(g\left(z,W\right)\right)\\ =\sigma \left(W_{2}\delta \left(W_{1}z\right)\right). \end{array}$$

**Figure 5:**
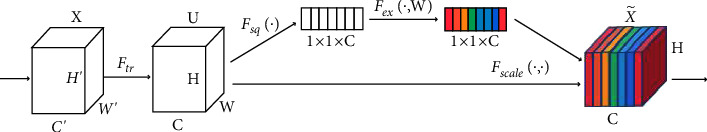
SENet module.

Its specific implementation is shown in Figure 5.

This paper adds an attention mechanism to filter out important information from a large amount of information. Through automatic learning, the neural network can get the importance of each channel in the feature map and make it more focused on some feature channels. In this way, we can increase the channel weights of the current task and suppress the feature channel weights that are useless for the current task, thereby improving the performance of the network model.

## 4. Experiment and Analysis

In this section, we introduce modules such as datasets and preprocessing, experimental environment and parameter setting, experimental model, experimental results, comparison with other algorithms, and ablation experiment.

### 4.1. Datasets and Preprocessing

This paper uses the CASIA-FaceV5, LFW, MTFL, CNBC, and ORL face datasets. The CASIA-FaceV5 Asian face dataset has 500 people, five pictures for each person, a total of 2500 pictures, and the picture size is 640 × 480. LFW has images of 5,749 individuals, with a total of 13,233 face images. Most people in the LFW dataset have only one face image, and very few people have two or more face images. And there are few black and white images in the LFW dataset. Each image in the LFW dataset is250 × 250 in size. The Multi-Task Facial Landmark (MTFL) has nearly 13,000 face images from the Internet. The CNBC dataset collects face photos of 200 people in different states (different expressions, outfits, hairstyles, etc.). The ORL dataset includes face pictures of 40 people, each person has ten face pictures, and each picture is a grayscale image with a resolution of 112 × 92. The datasets we used are all pictures with face accounting for at least two-thirds of the total.

**Figure 6:**
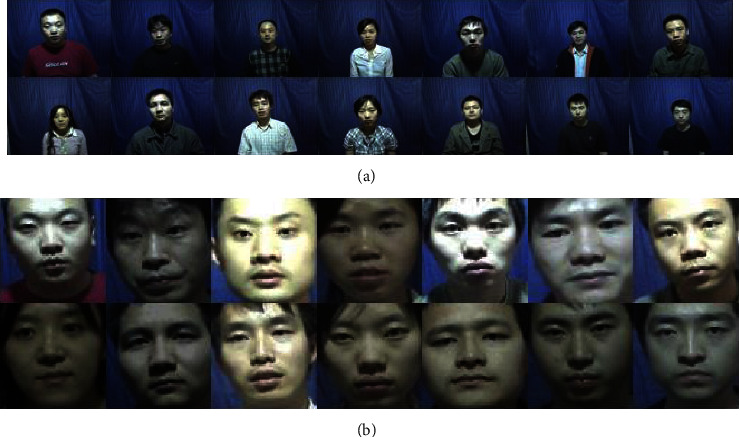
Partial face maps before and after preprocessing of the CASIA-FaceV5 dataset. (a) Partial face maps before preprocessing of the CASIA-FaceV5 dataset. (b) Partial face maps after preprocessing of the CASIA-FaceV5 dataset.

**Figure 7:**
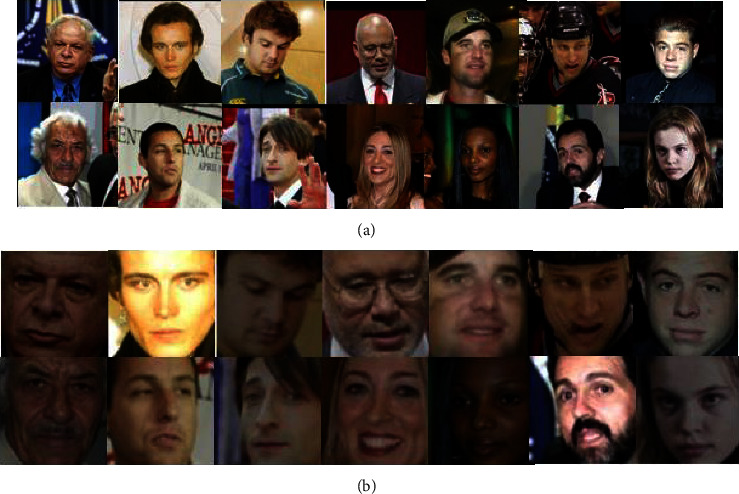
Partial face maps before and after preprocessing of the LFW dataset. (a) Partial face maps before preprocessing of the LFW dataset. (b) Partial face maps after preprocessing of the LFW dataset.

Before data training, the image needs to be preprocessed. Firstly, grayscale the image. Then, use the frontal_face_detector that comes with dlib to detect faces. Then, randomly adjust the brightness and contrast of the image to increase the sample diversity. Finally, resize the image to 64 × 64. The original data are divided into the training set and test set according to the probability of 3 : 7, and the face to be recognized and the labels of other faces are divided. This paper uses the CASIA-FaceV5, LFW, MTFL, CNBC, and ORL face datasets.  Figure 6(a) shows some face images before preprocessing on CASIA-FaceV5.  Figure 6(b) shows some face images after preprocessing on CASIA-FaceV5.  Figure 7(a) shows some face images before preprocessing on LFW.  Figure 7(b) shows some face images after preprocessing on LFW.

### 4.2. Experimental Environment and Parameter Settings

The experiments are all carried out on the platform of i7-6000CPU, 3.40 GHz, and 8G memory, using Python and NumPy for matrix calculation and using TensorFlow to develop the improved convolutional neural network at the back end.

In the parameter setting module, taking the LFW dataset as an example, we conducted experiments. The following two parameters, batch size and convolution kernel, are used to illustrate.

**Figure 8:**
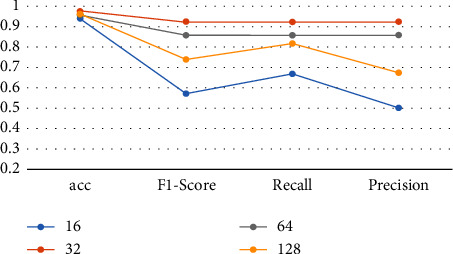
Evaluation indicators under different batch sizes.

Setting an appropriate batch size [19] can make the gradient descent direction more accurate and improve memory utilization and training speed. However, if the batch size is too small, the diversity of the samples will be lost, so the robustness of the trained neural network is not good. Too large a batch size will waste a lot of computing space, so you need to set a suitable batch size. The evaluation indicators under different batch sizes are shown in Table 1 and Figure 8.

It can be seen from Table 1 that the bold values perform best. When the batch size is 32, the recognition rate is the highest, so 32 is chosen as the batch training size.

The size of the convolution kernel determines [20] the output size. In theory, the smaller the convolution kernel, the better. But for particularly sparse data, when we use a relatively small convolution kernel, it may not be able to represent its features. Larger convolution kernels will lead to an increase in complexity. So, we need to choose the appropriate convolution kernel size.  Table 2 shows the evaluation metrics of faces under different sizes of convolution kernels.

From Table 2, it can be concluded that the bold values are the results with a 3 × 3 convolution kernel, making the face recognition best. Therefore, this paper chooses a 3 × 3 convolution kernel for experiments.

In CNN, batch size, learning rate, optimizer, pooling size, activation functions, loss functions, and kernel size are several important parameters. After many experiments, we have made the following settings for these parameters, as shown in Table 3.

### 4.3. Algorithm Model

**Figure 9:**
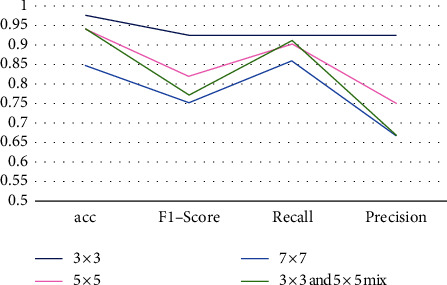
Evaluation indicators under different convolution kernels.

**Figure 10:**
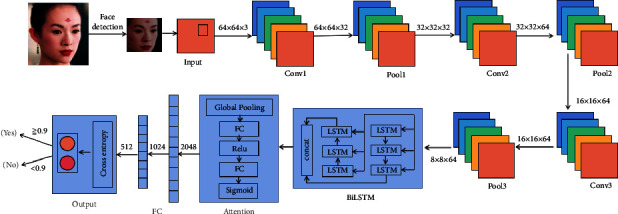
Algorithm model.

This paper uses the CASIA-FaceV5, LFW, MTFL, CNBC, and ORL datasets for sample training and prediction. The AB-FR model diagram is shown in Figure 9. Our input image size is 64 × 64 × 3. First, the features are extracted through three convolutional layers, and the number of channels is increased, but the image size is unchanged. The size of the convolution kernel we use is (3, 3), and the convolution stride is [1, 1, 1, 1]. Then, the input image is sampled through three pooling layers. This paper uses maximum pooling with a sampling size of 2 × 2. In this way, the length and width of the output feature map are half of the input feature map. Finally, add a dropout layer after each pooling layer to prevent overfitting. Besides, the features are input into BiLSTM to extract bidirectional information, and then the learned important features are extracted through the channel attention mechanism. Finally, in order to enhance the network's nonlinear ability and limit the network size, the network accesses two fully connected layers after the feature extraction layer extracts features. Each neuron in the fully connected layer is interconnected with all neurons in the previous layer, and it squashes the output of the image convolutional layer into a one-dimensional vector of 2 × 512. Its model is shown in Figure 10.

### 4.4. Experimental Results of AB-FR

**Figure 11:**
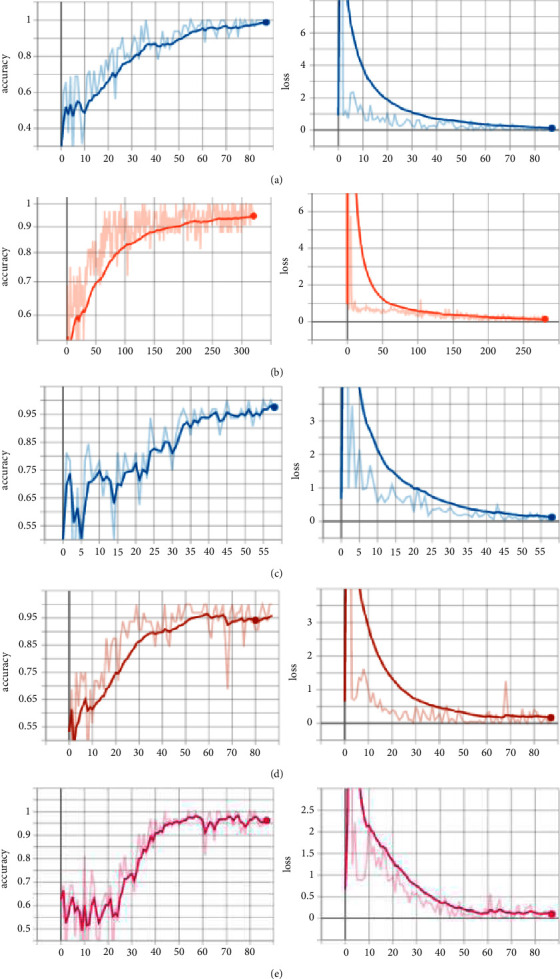
The performance of AB-FR on different datasets. (a) Acc and loss diagram of AB-FR on CASIA-FaceV5. (b) Acc and loss diagram of AB-FR on LFW. (c) Acc and loss diagram of AB-FR on MTFL. (d) Acc and loss diagram of AB-FR on CNBC. (e) Acc and loss diagram of AB-FR on ORL.

The performance of the AB-FR model on different datasets (CASIA-FaceV5, LFW, MTFL, CNBC, and ORL) is shown in Figure 11. It can be seen that the AB-FR model proposed in this paper has a high recognition rate on the CASIA-FaceV5 dataset. Because each person in CASIA-FaceV5 has five different pictures under the same background, there are some timing characteristics. The AB-FR proposed in this paper adds BiLSTM, fully considering the timing characteristics between pictures. Also, the attention mechanism is added to improve the extraction of dominant features, thereby improving the accuracy of face recognition. To avoid randomness, experiments are repeated ten times for each dataset. Specifically, each dataset is divided according to 3 : 7, in which one-seventh of the images are used as training samples, and one-third of the images are used as test samples. The best result of ten experiments is taken as the final result of this experiment.  Figure 11(a) shows the Acc and loss of AB-FR on CASIA-FaceV5.  Figure 11(b) shows the Acc and loss of AB-FR on LFW.  Figure 11(c) shows the Acc and loss of AB-FR on MTFL.  Figure 11(d) shows the Acc and loss of AB-FR on CNBC.  Figure 11(e) shows the Acc and loss of AB-FR on ORL.

**Figure 12:**
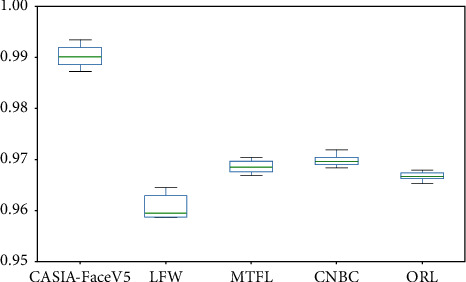
Box plot of the outputs of different datasets.

Figure 12 shows the output of 10 tests on different datasets as a box plot. As can be seen from the figure, the model proposed in this paper has a slightly higher result on the CASIA-FaceV5 dataset while a slightly lower result on the LFW dataset and a wider width of the box diagram. Because BiLSTM is added to the model proposed, the bidirectional sequential features of images are better considered. There are five different photos in CASIA-FaceV5 dataset, which are taken consecutively by different people in the same background, with certain temporal characteristics. LFW dataset, which contains more than 13,000 images from the Internet, has huge data and mixed backgrounds, and most of the images only have one photo. Therefore, the model proposed in this paper has a slightly better effect on the CASIA-FaceV5 dataset and a slightly worse effect on the LFW dataset.

In order to verify the statistical significance of the model presented in this paper, the Kruskal–Wallis test is used.  Table 4 shows the *P* value and Cohen's *f* value obtained from the Kruskal–Wallis test between AB-FR and the reference algorithms, where ^∗∗∗^, ^∗∗^, and ^∗^ represent the significance level of 1%, 5%, and 10%, respectively, and Cohen's *f* value represents the effect size. The distinguishing critical points of small, medium, and large effect sizes are 0.1, 0.25, and 0.40, respectively, which reflect the difference amplitude. As can be seen from the table, *P* values of test results are all less than 0.05, and Cohen's *f* values are all greater than 0.25. Therefore, the statistical results are significant, and there are moderate or large differences between AB-FR and other algorithms, indicating that the proposed AB-FR has a good performance.

### 4.5. Comparison with Other Algorithms

**Figure 13:**
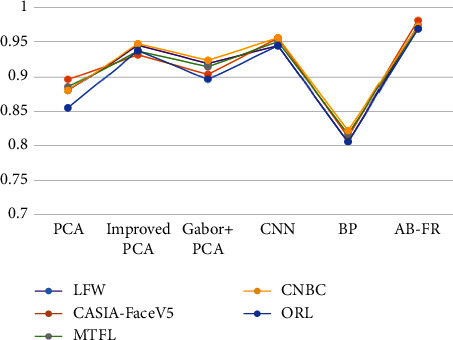
Face recognition rate graph of different algorithms.

The improved convolutional neural network algorithm proposed in this paper has a better recognition rate compared with other algorithms (PCA algorithm, improved PCA algorithm, Gabor + PCA algorithm, BP algorithm, traditional CNN algorithm, MobileNet, SCNN, and so on). The results are shown in Table 5 and the best results are the bold.  Figure 13 shows the accuracy comparison of the AB-FR algorithm proposed in this paper, the PCA algorithm, the improved PCA algorithm, the Gabor + PCA algorithm, the BP algorithm, and the traditional CNN algorithm. The ResNet + normalization + centerloss method proposed in the literature [10] focuses on normalization and loss functions, resulting in a higher recognition rate on the LFW and CNBC datasets. But it does not consider the temporal relationship between pictures, so the accuracy of CASIA-FaceV5 is not as good as the AB-FR proposed in this paper.

The experimental results show that the convolutional neural network structure designed in this paper has a good effect on the face recognition rate, and the results are better than those of the existing algorithms.

### 4.6. Ablation Experiment

**Figure 14:**
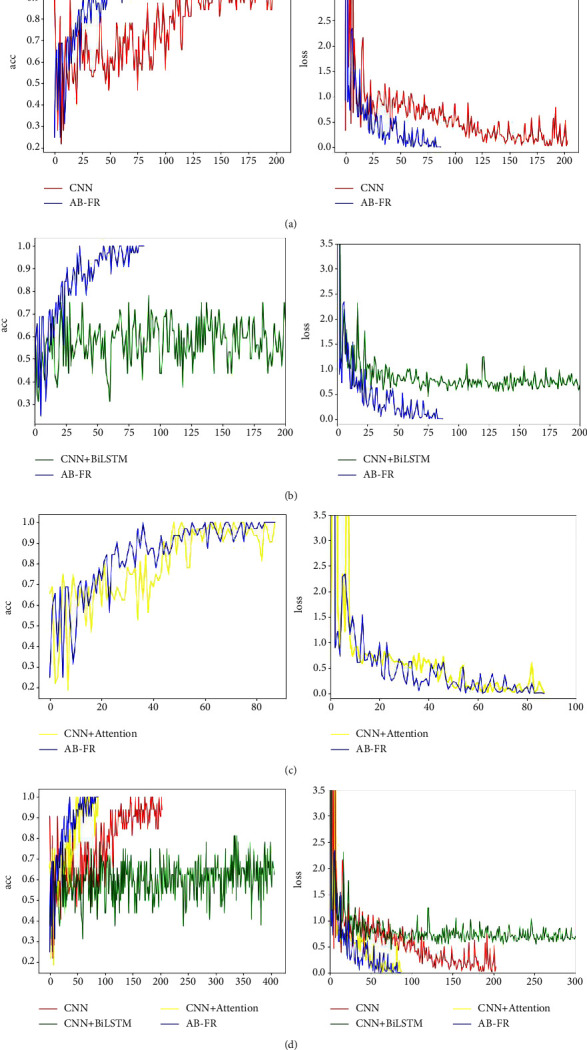
Visualization of the results of each model on CASIA-FaceV5. (a) Comparison of Acc and loss between CNN and AB-FR models. (b) Comparison of Acc and loss between CNN + BiLSTM and AB-FR models. (c) Comparison of Acc and loss between CNN + BiLSTM and AB-FR models. (d) Summary of Acc and loss of each model on CASIA-FaceV5.

**Figure 15:**
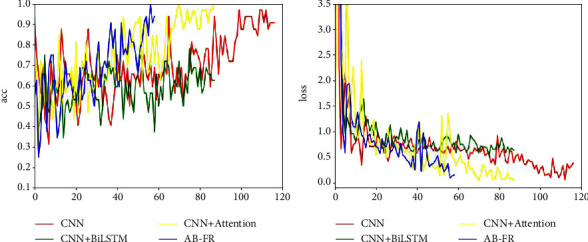
Visualization of the results of each model on LFW.

**Figure 16:**
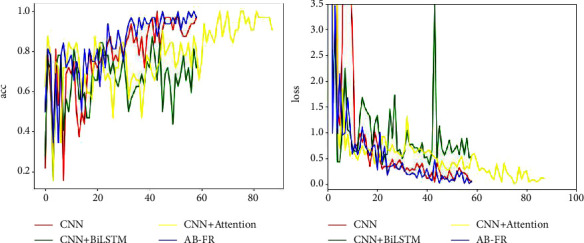
Visualization of the results of each model on MTFL.

**Figure 17:**
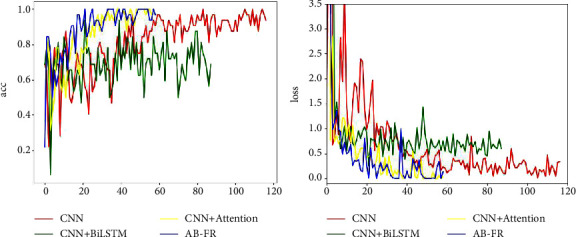
Visualization of the results of each model on CNBC.

**Figure 18:**
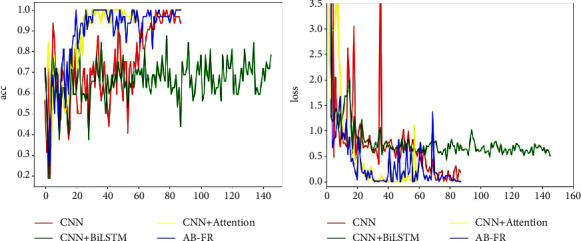
Visualization of the results of each model on ORL.

In order to verify the effectiveness of this model, an ablation experiment is performed, and it is decomposed into CNN, CNN + BiLSTM, and CNN + attention.  Figure 14 describes in detail the performance of CNN, CNN + BiLSTM, CNN + attention, and AB-FR on CASIA-FaceV5.  Figure 14(a) shows the Acc and loss charts of CNN on the CASIA-FaceV5 dataset.  Figure 14(b) shows the Acc and loss charts of CNN + BiLSTM on the CASIA-FaceV5 dataset.  Figure 14(c) shows the Acc and loss charts of CNN + attention on the CASIA-FaceV5 dataset.  Figure 14(d) shows the comparison of various models on the CASIA-FaceV5 dataset.  Figure 15 shows the comparison of the models on the LFW dataset.  Figure 16 shows the comparison of the models on the MTFL dataset.  Figure 17 shows the comparison of the models on the CNBC dataset.  Figure 18 shows the comparison of the models on the ORL dataset.  Table 6 indicates the results of the ablation experiment, and the best values are the bold. From Table 6 and Figures 14–18, it can be concluded that

1. Compared with the two models of CNN + BiLSTM and CNN + attention, CNN has a good recognition effect. But the accuracy is also slightly lower than that of the AB-FR model proposed in this paper. Also, it can be seen from Figure 11(a) that the CNN converges slowly within some time, showing a gentle trend, and there is some gradient disappearance problem.
2. The recognition rate of CNN + BiLSTM is low, which can only reach 68.5%. It can be seen from Figure 11(b) that the CNN + BiLSTM model cannot complete the task of face recognition very well.
3. The CNN + attention model is similar to the AB-FR model proposed in this paper, and the recognition effect and convergence speed are better than those of CNN and CNN + BiLSTM. But according to Figure 11(c), the recognition effect and convergence speed of CNN + attention are slightly inferior to the AB-FR model.
4. The results of the AB-FR model proposed in this paper are the best. It extracts salient features through the attention mechanism, avoids the loss of important features, reduces network training parameters, and improves training speed. With the addition of BiLSTM, the global and local features are comprehensively considered to obtain more accurate timing, so that the network model has a fast convergence speed and a high recognition rate.

## 5. Conclusions

This paper proposes a convolutional neural network face recognition method, AB-FR, based on BiLSTM and attention mechanism, aiming at the slow convergence speed of traditional CNN and the problems of occlusion and expression changes in face recognition in practical applications. The network uses CNN as the basic network structure, extracts key feature information, and assigns important weights by adding a channel attention mechanism. At the same time, BiLSTM is used to extract image time series features, which increases the reliability and effectiveness of image feature extraction. Also, we use cross-entropy as the loss function for supervised training to improve the accuracy of the model. The experimental results show that the method proposed in this paper has a significant effect on improving the convergence speed of the model without increasing too much computational overhead. It also has a good effect on improving the accuracy of face recognition. In the future, we will consider the face recognition problem in images containing multiple faces. Also, we will consider the impact of low image quality on face recognition after data compression and decoding.

## Figures and Tables

**Table 1:** Evaluation indicators under different batch sizes.

Batch size	Acc	*F*1-score	Recall	Precision
16	0.9375	0.5714	0.6667	0.5
32	**0.9729**	**0.9230**	**0.9230**	**0.9230**
64	0.9531	0.8571	0.8571	0.8571
128	0.9609	0.7381	0.8158	0.6739

The bold values indicate that batch size of 32 has the best experimental results, so the batch size used in this paper is 32

**Table 2:** Evaluation indicators under different convolution kernels.

Convolution kernel size	Acc	*F*1-score	Recall	Precision
3 × 3	**0.9729**	**0.9230**	**0.9230**	**0.9230**
5 × 5	0.9375	0.8181	0.9	0.75
7 × 7	0.8438	0.75	0.8571	0.6667
3 × 3 and 5 × 5 mix	0.9375	0.7692	0.9090	0.6667

**Table 3:** Related parameters.

Batch size	Learning rate	Optimizer	Pooling size	Activation functions	Loss functions	Kernel size
32	0.01	Adam	2 × 2	ReLu	Cross-entropy loss function	3 × 3

**Table 4:** Kruskal–Wallis test analysis results of AB-FR and the reference algorithms.

	BP	Improved PCA	PCA	Gabor + PCA	CNN	CNN + BiLSTM	CNN + attention
*P* value	0.025^*∗∗*^	0.009^*∗∗*^	0.009^*∗∗∗*^	0.009^*∗∗∗*^	0.009^*∗∗∗*^	0.008^*∗∗∗*^	0.028^*∗∗*^
Cohen's *f* value	0.629	0.44	0.481	0.47	0.379	0.498	0.345

**Table 5:** Face recognition rates of different algorithms.

Different algorithms	CASIA-FaceV5	LFW	MTFL	CNBC	ORL
ResNet + normalization + centerloss [10]	0.9535	**0.9814**	—	**0.9884**	—
MobileNet [11]	—	0.9553	—	—	—
Improved PCA [21]	0.931	0.9446	0.9362	0.9467	0.937
BP [22]	0.8126	0.823	0.8165	0.8217	0.805
PCA [22]	0.8954	0.8789	0.884	0.879	0.854
Gabor + PCA [23]	0.903	0.9186	0.914	0.9231	0.8956
CNN [24]	0.9548	0.959	0.9497	0.9552	0.944
PCANet_dense [25]	—	—	—	—	0.9563
KPCA + SVM [26]	—	—	—	—	0.9516
LBP + PCA + ELM [27]	—	—	—	—	0.91
HOG + LBP [28]	—	—	—	—	0.89
AB-FR	**0.9935**	0.9646	**0.9704**	0.9719	**0.9679**

**Table 6:** Evaluation results of each model in the ablation experiment.

Model	CASIA-FaceV5	LFW	MTFL	CNBC	ORL
CNN	0.938	0.944	0.9684	0.9663	0.9326
CNN + BiLSTM	0.699	0.685	0.713	0.7226	0.697
CNN + attention	0.943	0.939	0.9465	0.9698	0.9563
AB-FR	**0.9935**	**0.9646**	**0.9704**	**0.9719**	**0.9679**

## Data Availability

The datasets in the paper are all public datasets, and the acquisition methods are as follows. The CASIA-FaceV5 data used to support the findings of this study have been deposited in http://biometrics.idealtest.org/dbDetailForUser.do?id=9#/datasetDetail/9. The LFW data used to support the findings of this study have been deposited in http://vis-www.cs.umass.edu/lfw/index.html. The MTFL data used to support the findings of this study have been deposited in http://mmlab.ie.cuhk.edu.hk/projects/TCDCN.html. The CNBC data used to support the findings of this study have been deposited in https://github.com/260963172/CNBC/releases/tag/cnbc. The ORL data used to support the findings of this study have been deposited in https://github.com/260963172/ORL.
